# Supplementary material for: Impact of Combined Prebiotic Galacto-Oligosaccharides and Bifidobacterium breve-Derived Postbiotic on Gut Microbiota and HbA1c in Prediabetic Adults: A Double-Blind, Randomized, Placebo-Controlled Study
Source: Nutrients. 2024 Jul 10;16(14):2205. doi: 10.3390/nu16142205 (PMC11280236; doi:10.3390/nu16142205)
Supplement: Supplementary file 1 [file nutrients-16-02205-s001.zip › nutrients-3057805-supplementary.pdf]

Table S1. Biochemical analysis results of two groups at baseline and week 12. The data are expressed as Mean ± Standard Deviation (SD)

Blood Chemistries	Intervention (n=25)			Placebo (n=28)		
	Baseline	Week 12	*p*-value	Baseline	Week 12	*p*-value
HbA1c (%)	6.06±.36	5.68±.63	0.025	6.11±.42	6.02±.41	0.409
Fasting Plasma Glucose (mmol/l)	6.56±.77	6.01±.76	0.003	5.60±.53	5.61±.67	0.976
Total Cholesterol (mmol/l)	5.64±1.52	5.78±1.16	0.658	4.73±.51	5.17±.86	0.005
HDL-C (mmol/l)	1.43±.42	1.50±.39	0.280	1.14±.24	1.34±.33	0.003
LDL-C (mmol/l)	3.79±1.70	3.99±1.37	0.556	3.56±.92	3.54±.75	0.907
Triacylglycerol (mmol/l)	1.49±.83	1.36±.69	0.554	1.29±.84	1.53±.70	0.224
Total Protein (g/l)	58.0±7.27	59.81±3.77	0.325	60.33±5.98	59.52±4.60	0.587
Non-HDL-C (mmol/l)	3.94±1.63	4.36±1.43	0.131	3.60±.77	3.84±.78	0.096
Total Cholesterol: HDL Ratio	4.08±1.22	4.08±1.16	0.963	4.30±.79	4.06±1.04	0.245

Data were analysed using the Paired Samples t-Test. *p* value less than .05 (2-sided *P* value) was considered statistically significant.

Variable	Baseline	Post-Intervention	Treatment	Time	Interaction
LBP, ug/mL					
Intervention	14.8 ± 0.3	12.4 ± 0.9	0.732	0.002*	0.207
Control	18.7 ±	15.4 ± 1.1		0.6	

[1] Values are mean ± SEM.
[2]*P* values calculated using Repeated-Measures ANOVA. **P* ≤ 0.05 was considered statistically significant,
Abbreviation: LBP, lipopolysaccharide-binding protein
